# Гепатопульмональный синдром — редкая манифестация цирроза печени у пациентки с диэнцефальным ­ожирением после удаления краниофарингиомы

**DOI:** 10.14341/probl12723

**Published:** 2021-10-03

**Authors:** Н. А. Мазеркина, А. Н. Саватеев, С. К. Горелышев, С. А. Маряшев, С. А. Береговская, А. Н. Коновалов

**Affiliations:** Национальный медицинский исследовательский центр нейрохирургии им. Н.Н. Бурденко; АО «Деловой центр нейрохирургии»; Национальный медицинский исследовательский центр нейрохирургии им. Н.Н. Бурденко; Национальный медицинский исследовательский центр нейрохирургии им. Н.Н. Бурденко; Национальный медицинский исследовательский центр терапии и профилактической медицины; Национальный медицинский исследовательский центр нейрохирургии им. Н.Н. Бурденко

**Keywords:** краниофарингиома, гипопитуитаризм, диэнцефальное ожирение, цирроз печени, гепатопульмональный синдром

## Abstract

В статье описан клинический случай пациентки 15 лет, у которой в результате удаления краниофарингиомы, помимо несахарного диабета и пангипопитуитаризма, развилось диэнцефальное ожирение, приведшее к формированию цирроза печени. В данном случае патология печени манифестировала гепатопульмональным синдромом, характеризующимся гипоксемией, причиной которой является патологическое расширение сосудов легких, приводящее к шунтированию крови у пациентов с поражением печени. Несмотря на отсутствие рецидива опухоли после удаления и радиохирургического лечения, а также адекватную заместительную гормональную терапию, пациентка скончалась через 9 лет после операции. Заместительная терапия гормоном роста у пациентов с гипопитуитаризмом и жировым гепатозом может уменьшить аккумуляцию липидов в печени и снизить риск развития цирроза.

## Abstract (en)

We describe a 15-year girl, who developed panhypopituitarism and diencephalic obesity after surgical excision of craniopharyngioma, followed by nonalcoholic fatty liver disease and cirrhosis 5 years after surgery. Cirrhosis in this case manifested by hypoxia due to hepatopulmonary syndrome, and despite cure of craniopharyngioma by surgery and radiosurgery treatment and adequate hormonal substitution therapy patient died 9 years after surgery. Growth hormone substitutional therapy in patients with hypopituitarism, and steatohepatitis may decrease liver triglyceride accumulation and prevent end-stage liver disease.

## АКТУАЛЬНОСТЬ

Краниофарингиомы (КФ) — доброкачественные эпителиальные опухоли дизэмбриогенетического происхождения, растущие из остатков краниофарингеального хода, который в период эмбрионального развития соединяет полость ротовой трубки с аденогипофизом. Они составляют около 6–9% всех новообразований головного мозга у детей, являясь самыми распространенными опухолями гипоталамо-гипофизарной системы [1]. Эмбриогенез и локализация опухоли обусловливают высокую частоту эндокринно-обменных нарушений у этих больных. К ним относятся дефицит гормонов передней доли гипофиза или гипопитуитаризм (недостаточность гормона роста (ГР), вторичный гипотиреоз, гипокортицизм, гипогонадизм), несахарный диабет (НД), ожирение, гиперпролактинемия. Помимо этого, заболевание может проявляться зрительными расстройствами, симптомами повышения внутричерепного давления. Учитывая доброкачественный характер опухоли, основным методом ее лечения является хирургическое удаление.

Даже частичное удаление опухоли, как правило, приводит к нарастанию эндокринного дефицита [2]. Опухоль локализуется вблизи жизненно важных структур (зрительных путей, гипофиза, сосудов виллизиева круга, диэнцефальной области), поэтому агрессивная хирургическая тактика может способствовать повреждению этих структур, приводя к нарастанию частоты зрительных, диэнцефальных нарушений [3], НД [4] и дефицита гормонов передней доли гипофиза [5]. Особой проблемой является диэнцефальное ожирение, поражающее, по данным литературы, до 50% больных с КФ после хирургического лечения [6]. У больных диэнцефальным ожирением развивается ряд метаболических и сердечно-сосудистых осложнений, что приводит к повышению инвалидизации и смертности в данной группе пациентов [1][7–9]

Одним из последствий диэнцефального ожирения является неалкогольная жировая болезнь печени (НАЖБП), которая может приводить к циррозу печени.

## ОПИСАНИЕ СЛУЧАЯ

У пациентки А.Ш. с 13 лет отмечены замедление роста и остановка полового созревания. В 15 лет появились головные боли, к которым через несколько месяцев присоединились рвота, снижение остроты зрения до 0,4 на оба глаза, правосторонняя гомонимная гемианопсия. При МРТ выявлена мультикистозная интраэкстравентрикулярная КФ (рис. 1А), окклюзионная гидроцефалия. В январе 2008 г. (15,9 года) поступила в НМИЦ нейрохирургии им. академика Н.Н. Бурденко для хирургического лечения.

Результаты физикального, лабораторного и инструментального исследования

По данным обследования: до операции рост 145 см (SDS роста -2,7, рост матери 156 см, отца 175 см), вес 38 кг (индекс массы тела (ИМТ) 18,1 кг/м2). Половое развитие по Таннеру 2, менструаций не было. В анализе крови до операции выявлено снижение инсулиноподобного фактора роста 1 (ИФР-1) и половых гормонов, остальные показатели в пределах нормы (табл. 1). Диагностирован дефицит ГР, вторичный гипогонадизм.

Лечение.

Больная была оперирована — проведено субтотальное удаление опухоли комбинированным доступом (транскаллезным и птериональным) (рис. 1Б). После операции развился НД, назначен десмопрессин. Ранний послеоперационный период протекал тяжело — отмечались электролитные расстройства (уровень натрия колебался в пределах 125–155 нМ/л), в течение 2 нед проводилась интенсивная терапия в реанимационном отделении. После стабилизации показателей электролитного обмена девочка выписана.

Исход и результаты последующего наблюдения.

При обследовании через 2 мес после операции выявлено снижение уровней свободного тироксина (Т4) и кортизола в сыворотке крови (см. табл. 1), диагностирован пангипопитуитаризм, назначена заместительная терапия (левотироксин, гидрокортизон). Через 6 мес после операции к терапии добавлены половые гормоны (препараты эстрадиола и прогестерона). Также после операции пациентка начала быстро прибавлять в весе, невзирая на соблюдение гипокалорийной диеты (через 2 мес после операции масса тела увеличилась с 38 до 52 кг) (рис. 2).

В феврале 2010 г. (через 2 года после хирургического лечения) при очередной контрольной МРТ выявлен рецидив опухоли небольших размеров, расположенный эндосупраселлярно (рис. 1В). Проведено стереотаксическое радиохирургическое лечение на аппарате Гамма-нож с предписанной дозой 12 Гр, предписанной изодозой 50% на мишень объемом 1,1 см3. В динамике после облучения остаток опухоли уменьшился (рис. 1Г).

**Figure:**
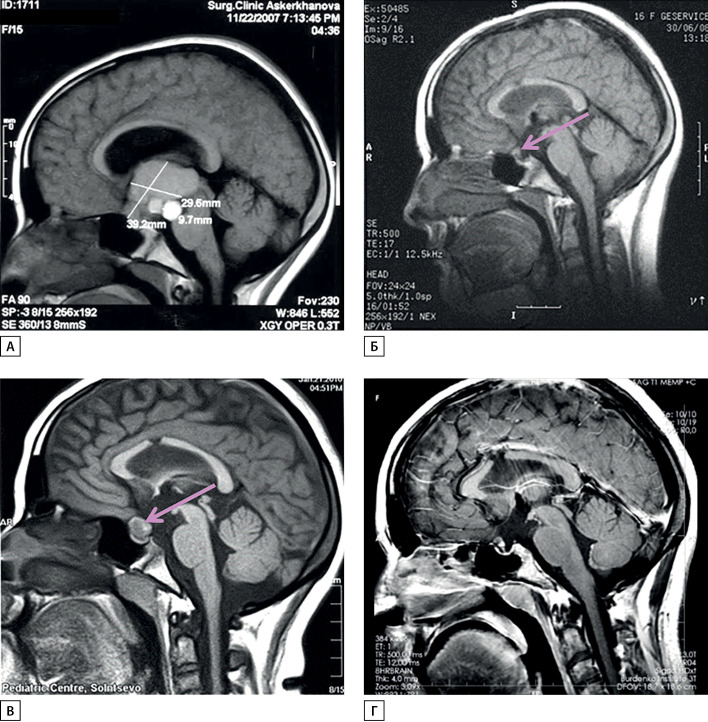
Рисунок 1. МРТ головного мозга пациентки в динамике.А — до операции; Б — через 6 мес после субтотального удаления краниофарингиомы (определяется небольшой остаток опухоли в хиазмально-селлярной области); В — рецидив опухоли (увеличение остатка) через 2 года после удаления перед радиохирургическим лечением; Г — уменьшение остатка опухоли через год после радиохирургического лечения.

**Table:** Таблица 1. Динамика показателей уровней гормонов у пациентки до, через 1 мес и через 5 лет после операции*

Показатель(референсные значения)	До операции	Через 1 мес после операции	Через 5 лет после операции при манифестации ГПС
ТТГ, мЕд/л (0,4–4,0)	2,4	4,3	0,01
Свободный Т4, пмоль/л (11–25)	21,5	7,2	14,3
Кортизол, нмоль/л (140–640)	563	93	120
Пролактин, мЕд/л (40–550)	327	912	340
ЛГ, Ед/л	0,4	0,2	<0,1
ФСГ, Ед/л	1,7	0,8	<0,1
Эстрадиол, пмоль/л (100–905)	<70	<70	99
ИФР-1, нг/мл (120–480)	67	96	<25
Инсулин, мкЕ/мл (2,7–10,5)	8,8	42	33
НОМА (до 3)	1,96	11,6	7,2

**Figure:**
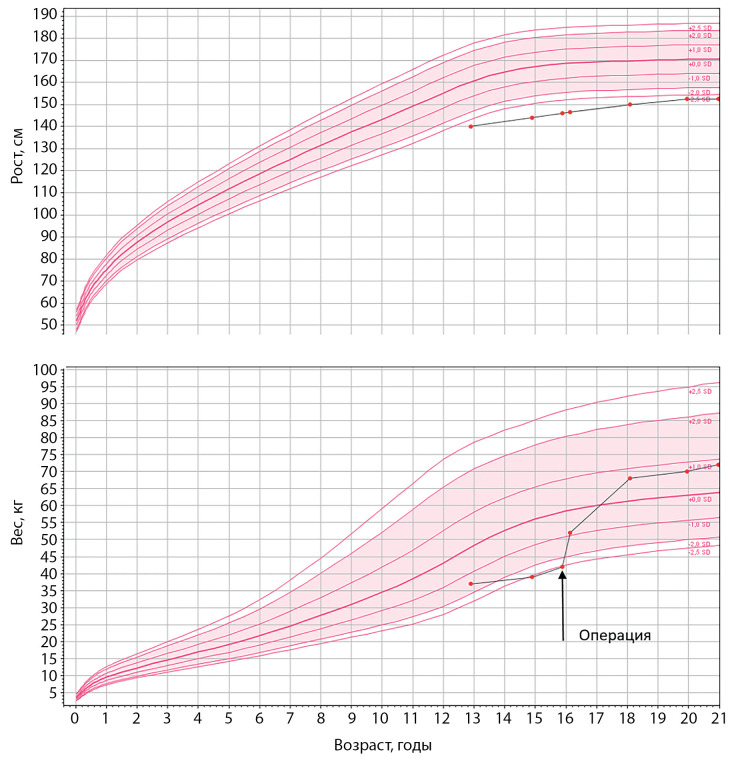
Рисунок 2. Динамика роста и веса пациентки до и после операции.

По данным обследования, через 2 года после операции перед радиохирургическим лечением беспокоят слабость, отечность (преимущественно лица, верхних и нижних конечностей), эпизоды гипертермии до 39,5°С без катаральных явлений и других признаков инфекции, приступы тошноты, изжоги (при эзофагогастродуоденоскопии выявлен эрозивный гастродуоденит), периодически образование трофических язв на пальцах ног. Принимает левотироксин 75 мкг, гидрокортизон 7,5 мг в сутки, десмопрессин интраназально 40 мкг в сутки, 17-β-эстрадиол трансдермально 1 мг в сутки, микроионизированный прогестерон 200 мг с 16-го дня цикла 10 дней, урсодезоксихолевую кислоту 500 мг в сутки, метформин 1500 мг в сутки. Антропометрические характеристики: рост 150 см, вес 68 кг (ИМТ 30,2 кг/м2), ЧСС 92 в 1 мин, АД 100/70 мм рт. ст. Менструации на фоне заместительной терапии регулярные. В анализе крови отмечалась тенденция к гипохромной анемии (гемоглобин 114 г/л), умеренное повышение печеночных ферментов (АЛТ 59 Ед/л, АСТ 65 Ед/л, ГГТ 128 Ед/л), повышение уровня глюкозы натощак до 6,2 мМоль/л при нормальном уровне гликированного гемоглобина (табл. 2), гипернатриемия 155  мМоль/л (норма 135–145). По данным анализа крови на гормоны пациентка скомпенсирована по гормональным показателям, отмечается повышение индекса инсулинорезистентности (НОМА) до 11,6 (см. табл. 1). На УЗИ органов брюшной полости были отмечены увеличение печени, признаки жирового гепатоза.

**Table:** Таблица 2. Динамика показателей общего и биохимического анализа крови у пациентки до, через 2 года и через 5 лет после операции

Показатель (референсные значения)	До операции	Через 2 года после операции	Через 5 лет после операции при манифестации ГПС
Гемоглобин, г/л (120–140)	140	114	178
Эритроциты (3,9–7,7 ×1012)	4,85	3,9	6,8
Цветовой показатель (0,85–1,05)	-	0,78	0,77
MCV (84–96), фл	86,4	79	77
Тромбоциты (150–400)	352	420	136
Глюкоза, мМоль/л (3,3–5,5)	5.0	6,2	4,9
АЛТ, Ед/л, (до 40)	30	59	38
АСТ, Ед/л (до 40)	21	69	32
ГГТ, Ед/л (до 40)	40	128	80
Билирубин общий, мкМоль/л (5–20)	11,5	20,5	31
Общий белок, г/л (65–85)	82	76	80
Альбумин, г/л (35–50)	49	44	38
Гликированный гемоглобин, % (до 6,1)	4,7	5,5	6,1

Через 4 года после операции (с марта 2012 г.) усилились отеки (по утрам в основном лица и верхних конечностей, вечером — нижних конечностей), появился акроцианоз, с осени 2012 г. появились синкопальные состояния частотой 1 раз в 2–3 нед. По данным обследования в феврале 2013 г. (через 5 лет после операции) ИМТ увеличился до 31,6 кг/м2. Отмечается одышка, усиливающаяся в вертикальном положении (платипноэ), цианоз носогубного треугольника и ногтевых фаланг, характерные изменения фаланг пальцев по типу барабанных палочек (рис. 3). ЧСС 98 в 1 мин, АД 110/70 мм рт. ст. В общем анализе крови было выявлено повышение уровня гемоглобина до 178 г/л, эритроцитоз до 6,8×1012, снижение уровня тромбоцитов до 136×109 (табл. 2). В биохимическом анализе крови отмечалось повышение уровня билирубина до 31 мкМоль/л, повышение ГГТ до 80 Ед/л (см. табл. 2). По данным эхокардиографии признаков легочной гипертензии не отмечено, гемодинамически незначимое провисание митрального клапана. Суточное мониторирование ЭКГ и АД без патологии, мультиспиральная компьютерная томография (МСКТ) органов грудной клетки без патологии. Учитывая признаки хронической гипоксии неясного генеза, в апреле 2013 г. обследована в НМИЦ терапии и профилактической медицины МЗ РФ. При пульсоксиметрии сидя выявлена существенная разница в показателях сатурации крови верхних и нижних конечностей: 65 и 95% соответственно. В горизонтальном положении сатурация крови на верхних и нижних конечностях составляла 70%, то есть отмечалась ортодексия — снижение сатурации при переходе в вертикальное положение. По данным проведенного дополнительного обследования в анализе кислотно-щелочного состояния артериальной крови выявлено снижение парциального давления кислорода (рО2) до 26 мм рт. ст. (норма 75–100). УЗИ органов брюшной полости выявило диффузное увеличение печени и селезенки, признаки жирового гепатоза. При МСКТ органов средостения и брюшной полости выявлена гепатоспленомегалия, расширение портальной вены. При пункции костного мозга патологии не выявлено (пунктат гипоклеточный, мегакариоциты в достаточном количестве).

**Figure:**
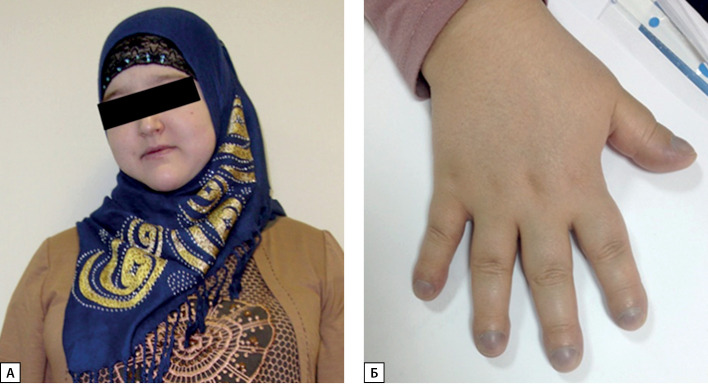
Рисунок 3. Внешний вид пациентки.А — цианоз носогубного треугольника; Б — изменения ногтевых фаланг по типу барабанных палочек и акроцианоз.

При исследовании функции внешнего дыхания (ФВД) выявлены рестриктивные изменения: снижение общей емкости легких (TLC) до 79%, жизненной емкости легких (VC) до 66%, форсированной жизненной емкости легких (FVC) до 66%, выраженное снижение диффузионной способности легких (DLCO) до 30,5%.

Таким образом, у пациентки отмечались признаки хронической гипоксемии: синкопальные состояния, цианоз, изменения ногтевых фаланг и ногтей, компенсаторная эритремия, снижение рО2 артериальной крови. Как причины этого состояния исключены патология крови, сердца, легких и дыхательных путей. Учитывая гепатомегалию с признаками портальной гипертензии, гиперспленизм (увеличение селезенки и тромбоцитопения), был заподозрен гепатопульмональный синдром (ГПС), при котором отмечается расширение легочных сосудов и шунтирование крови в них. Характерными клиническими симптомами ГПС являются платипноэ и ортодексия, которые отмечались у пациентки. Для визуализации сброса крови была проведена эхокардиография с пузырьковым контрастированием (Bubble test, проба с ажитированным физиологическим раствором) — после контрастирования правых отделов сердца на 4-м сокращении было выявлено также контрастирование левых отделов, что свидетельствует о наличии внутрилегочных шунтов легочных артерий с легочными венами и вследствие этого нарушении газообмена в легких.

Пациентка дополнительно обследована гепатологом. Анализ на гепатиты отрицательный. Антинуклеарные и антимитохондриальные антитела отрицательные. Церулоплазмин 0,33 мг/л (норма 0,2–0,6).

Эластография печени: зарегистрировано 10 валидных изменений печеночной ткани. Медиана значений составила 34,3 кПа (k25–k75 = 8,6 кПа), что соответствует степени фиброза F4 по шкале METAVIR.

Дуплексное сканирование нижней полой вены и вен портальной системы с цветовым картированием кровотока: нижняя полая вена диаметром до 14–15 мм, на вдох коллабирует менее 50%. На фоне признаков гепатоспленомегалии и выраженных диффузных изменений печени (фиброза) воротная вена расширена до 15–16 мм, селезеночная — до 11 мм. Кровоток по воротной вене до 11–12,5 см/с, гепатопетального типа с низкой фазностью. Ветви воротной вены минимально расширены. Заключение: эхо-признаки гепатоспленомегалии, фибросклеротических изменений паренхимы печени, синдрома портальной гипертензии. Косвенные признаки легочной гипертензии.

Был диагностирован фиброз печени 4-й стадии, что соответствует циррозу печени по рекомендациям Европейской ассоциации по изучению болезней печени (European Association Study Liver Disease, EASL) 2016 г. [10] с портальной гипертензией, ГПС тяжелой степени. Пациентке назначена оксигенотерапия, на фоне которой уменьшилась одышка, регрессировали приступы утраты сознания, показатели оксигенации крови по данным пульсоксиметрии на верхних и нижних конечностях увеличились до 89% сидя и 93% лежа. Рекомендован курсовой прием гепатопротекторов. Также рекомендована биопсия печени, от которой родственники отказались. Несмотря на отсутствие выраженной печеночно-клеточной недостаточности, учитывая наличие ГПС, рекомендовано решение вопроса о трансплантации печени.

Пациентка продолжала получать заместительную и симптоматическую терапию, с осени 2016 г. ее состояние стало быстро ухудшаться, развилась полиорганная недостаточность, и в феврале 2017 г. в возрасте 22 лет она скончалась. При этом в течение 7 лет после окончания комбинированного лечения (удаление опухоли и радиохирургия) рецидива КФ выявлено не было. Таким образом, причиной смерти стала не прогрессия опухоли, а цирроз печени, развившийся на фоне серьезных метаболических нарушений в результате травмы гипоталамуса.

## ОБСУЖДЕНИЕ

КФ — это доброкачественная опухоль, поэтому основным методом лечения данной патологии является ее удаление. Долгие годы ведущие мировые нейрохирурги считали оптимальным методом лечения максимально радикальное удаление, публикуя многочисленные серии наблюдений, в том числе у детей [7][9]. Основное внимание уделялось сохранности зрения и других неврологических функций. Эндокринные нарушения при удалении КФ считались неизбежными и приемлемыми, причем исследователи в основном концентрировались на гормональном дефиците, а не на диэнцефальных проблемах, которые развиваются вследствие травмы гипоталамуса.

Гипоталамус, располагающийся в диэнцефальной области и имеющий объем всего 4 мл, является важнейшим центром регуляции энергетического гомеостаза. Помимо синтеза пептидов, стимулирующих секрецию гормонов гипофиза, он содержит группы ядер, играющих важную роль в синхронизации биологических и циркадных ритмов, регулирует баланс симпатической и парасимпатической нервной системы. Гипоталамические ядра взаимодействуют со многими гормонами и пептидами (включая лептин, инсулин, пептиды семейства игрек, грелин), а также глюкозой, жирными кислотами, аминокислотами, регулируя потребление пищи и расход энергии. Основными гипоталамическими регуляторами энергообмена являются проопиомеланокортин (ПОМК), нейропептид Y и агутиподобный пептид.

В 1996 г. впервые J. de Vile и соавт. [1] показали, что степень инвалидизации пациентов после удаления КФ зависит от возраста и наличия диэнцефальных нарушений, основным внешним проявлением которых является диэнцефальное ожирение. Данное ожирение сопровождается слабостью, снижением физической активности, снижением основного обмена, повышенным аппетитом, дисбалансом вегетативной нервной системы (повышением парасимпатического и снижением симпатического тонуса) [11]. У нашей пациентки после операции по удалению опухоли, помимо пангипопитуитаризма и НД, развилось выраженное диэнцефальное ожирение. Такое ожирение также сопровождается гиперсекрецией инсулина и инсулинорезистентностью, могут развиваться нарушения углеводного обмена (у нашей пациентки нарушение гликемии натощак на фоне повышенного НОМА). К сожалению, при данном виде ожирения модификация образа жизни (правильное питание и физические нагрузки) малоэффективна.

Гипоталамус также является важным центром регуляции циркадных ритмов, температурного баланса, водного и электролитного обмена. У нашей пациентки, помимо ожирения, наблюдались другие последствия травмы диэнцефальной области: эпизоды гипертермии, вероятно, центрального генеза, склонность к гипернатриемии, обусловленная нарушением чувства жажды, нарушение трофики тканей (эрозивный гастрит, трофические язвы пальцев ног).

Выраженность диэнцефальных нарушений зависит от степени травмы гипоталамуса. Благодаря работе J. De Vile и соавт. к мировому медицинскому сообществу постепенно стало приходить понимание, что поражение диэнцефальной области приводит к необратимым последствиям и в отличие от дефицита гормонов гипофиза не корригируется медикаментозно. Было предложено учитывать факторы риска травмы гипоталамуса, наиболее важным из которых является оценка предоперационных МРТ. Дальнейшие исследования продемонстрировали, что степень поражения гипоталамуса по данным МРТ у больных с КФ напрямую коррелирует не только с риском развития ожирения, но и с тяжестью послеоперационного периода, развитием психоэмоциональных и когнитивных нарушений [12]. Шкала оценки инвазии опухолью гипоталамуса по МРТ была развита другими нейрохирургами [13], было выделено три степени вовлечения гипоталамуса в опухоль до операции. При наличии инвазии до операции рекомендовано намеренно нерадикально удалять опухоль, чтобы избежать тяжелой инвалидизации пациентов.

Это привело к тому, что во многих ведущих клиниках хирургия стала более консервативной. Например, по данным ведущей клиники Роттердама, частота тотального удаления КФ снизилась с 22% в 1980–1989 гг. до 12% после 2010 г., а частота субтотального удаления увеличилась с 30% в 1980–1989 гг. до 71% после 2010 г. [14]. Такая тактика привела к снижению частоты морбидного ожирения и тяжелых неврологических нарушений.

При нерадикальном удалении КФ значительно увеличивается риск рецидива опухоли, поэтому в таких случаях может быть рекомендовано проведение лучевой терапии (радиохирургического лечения) после операции. У нашей пациентки через 2 года после операции отмечался продолженный рост опухоли, который был успешно пролечен с помощью радиохирургического метода на аппарате Гамма-нож.

Работа E. Elowe-Gruau и соавт., обобщившая результаты одного центра Necker (Париж, Франция) показала, что более консервативная хирургическая тактика, направленная на снижение риска травмы гипоталамуса с последующим облучением, достоверно снижает риск тяжелого ожирения, не увеличивая риск рецидива опухоли [4]. Исследователи сравнили историческую когорту операций с 1985 по 2002 гг. (n=37) более агрессивной хирургии с проспективной когортой детей (n=38), оперированных между 2002 и 2010 гг. В проспективной когорте использовалась более консервативная тактика при риске травмы гипоталамуса. Тяжелое ожирение достоверно чаще развивалось в исторической когорте (54 и 28% соответственно). Также, несмотря на использование лучевой терапии, которую традиционно связывают с нарастанием гипопитуитаризма, частота гипогонадизма, гипокортицизма и НД была достоверно ниже в проспективной когорте.

В когортных исследованиях было показано, что смертность больных с КФ значительно выше, чем в популяции, — относительный риск составляет от 2,88 до 9,28 [2]. При этом риск сердечно-сосудистой смерти повышается в 3–19 раз, женщины имеют более высокий риск, чем мужчины [15]. Диэнцефальное ожирение является серьезным фактором риска сердечно-сосудистой смерти, так, в частности, у пациентов с КФ чаще, чем в популяции, встречается обструктивное апноэ [16]. Помимо ожирения, гипопитуитаризм также может быть причиной повышения сердечно-сосудистой смертности — это некомпенсированный дефицит ГР, терапия препаратами гидрокортизона в высоких дозах (в среднем 15–30 мг в сутки в исторических когортах), а также неадекватная терапия половыми гормонами у женщин (с применением контрацептивных препаратов или отсутствие терапии половыми гормонами) [17]. Другие исследования также показывают повышение смертности от острых инфекций и вторичных опухолей (при применении лучевой терапии) [14].

У обсуждаемой нами пациентки на фоне диэнцефального ожирения развилась НАЖБП, которая в настоящее время является самой распространенной болезнью печени в развитых странах. Распространенность НАЖБП составляет около 80% среди лиц с ожирением и 16% среди лиц с нормальным ИМТ [18]. НАЖБП связана с метаболическим синдромом, абдоминальным ожирением, синдромом поликистозных яичников.

НАЖБП — часто встречающееся осложнение гипоталамического ожирения, у взрослых пациентов с КФ, поражающей гипоталамическую область, ее частота составляет около 50% [19], по данным другого исследования, в общей популяции взрослых пациентов, лечившихся в детстве по поводу КФ, даже выше — около 68% [20]. По данным этого исследования, наличие НАЖБП коррелировало не с ИМТ пациента, а с количеством жировой ткани и индексом инсулинорезистентности НОМА. Развивается она в среднем через 4–5 лет после операции, как и в нашем клиническом наблюдении. Высокая частота НАЖБП у пациентов с КФ говорит о важности мониторирования печеночных показателей.

Цирроз печени — это нередкое осложнение у пациентов с НАЖБП, однако ГПС — это малоизвестное осложнение заболеваний печени, основным проявлением которого является гипоксемия на фоне заболеваний печени при отсутствии кардиореспираторных заболеваний. У описанной пациентки отмечалась характерная клиническая картина гипоксемии на фоне ГПС: акроцианоз, изменения ногтевых фаланг по типу барабанных палочек и ногтей по типу часовых стекол, синкопальные состояния, компенсаторная эритремия, снижение оксигенации крови.

Причиной этого состояния является нарушение оксигенации крови в легких, вызванное дилатацией сосудов легких и реже — развитием плевральных и легочных артерировенозных шунтов на фоне портальной гипертензии [10]. Впервые гипоксемия при печеночной патологии была описана Flückige еще в 1884 г. Патогенез этого состояния до конца не ясен, по некоторым данным, вазодилатация легочных сосудов связана с неадекватным синтезом или метаболизмом в поврежденной печени вазоактивных субстанций, важную роль среди которых играет оксид азота [21].

После трансплантации печени восстанавливается нормальный газообмен в легких, поэтому, согласно клиническим рекомендациям EASL, ГПС является показанием к трансплантации печени, независимо от степени ее поражения [10].

В литературе есть несколько сообщений о развитии ГПС у больных с гипопитуитаризмом и гипоталамическим ожирением [22], в том числе после удаления КФ [23]. НАЖБП течет более агрессивно у пациентов с гипопитуитаризмом и поражением диэнцефальной области, чем в общей популяции. Одна из причин этого — дефицит ГР. Дефицит ГР связан с дислипидемией и увеличением количества внутрипеченочного жира, при акромегалии, наоборот, у пациентов отмечается снижение содержания висцерального и печеночного жира [24].

Наша пациентка получала адекватную заместительную терапию гипопитуитаризма, за исключением ГР. В случаях НАЖБП на фоне дефицита терапия ГР может оказать благоприятный эффект, уменьшая степень накопления липидов в печени. В частности, был описан пациент с герминативно-клеточной опухолью и гипоталамическим ожирением, перенесший трансплантацию печени в связи с циррозом печени, у которого заместительная терапия ГР после трансплантации привела к нормализации печеночных ферментов и снижению содержания жиров в печени по данным КТ [22].

## ЗАКЛЮЧЕНИЕ

В данном случае рассмотрено клиническое наблюдение пациентки с КФ, у которой после удаления опухоли в результате травмы диэнцефальной области развились ожирение и цирроз печени на фоне НАЖБП, манифестировавшей с ГПС. Улучшить прогноз у пациентов с КФ поможет мультидисциплинарный подход с оценкой риска радикального удаления опухоли на дооперационном этапе. Заместительная терапия дефицита ГР, возможно, снизит риск развития НАЖБП у пациентов с диэнцефальным ожирением, получавших лечение опухолей мозга.

## ДОПОЛНИТЕЛЬНАЯ ИНФОРМАЦИЯ

Источники финансирования. Работа выполнена по инициативе авторов без привлечения финансирования.

Конфликт интересов. Авторы декларируют отсутствие явных и потенциальных конфликтов интересов, связанных с содержанием настоящей статьи.

Участие авторов. Мазеркина Н.А. — сбор и обработка материала, интерпретация результатов, написание текста, подготовка рукописи; Саватеев А.Н. — сбор и обработка материала, подготовка рукописи, интерпретация результатов, написание текста; Горелышев С.К. — критическая интерпретация результатов, внесение существенной правки, одобрение финальной версии; Маряшев С.А. — сбор и обработка материала, интерпретация результатов, написание текста, подготовка рукописи; Береговская С.А. — сбор и обработка материала, интерпретация результатов, внесение существенной правки; Коновалов А.Н. — критическая интерпретация результатов, внесение существенной правки, одобрение финальной версии. Все авторы одобрили финальную версию статьи перед публикацией, выразили согласие нести ответственность за все аспекты работы, подразумевающую надлежащее изучение и решение вопросов, связанных с точностью или добросовестностью любой части работы.

Согласие пациента. Законный представитель пациента добровольно подписал информированное согласие на публикацию персональной медицинской информации в обезличенной форме в журнале.
